# *In vitro* evaluation of different varieties of maize fodder for their methane generation potential and digestibility with goat rumen liquor

**DOI:** 10.14202/vetworld.2016.1209-1213

**Published:** 2016-11-08

**Authors:** Shalini Vaswani, Ravindra Kumar, Vinod Kumar, Debashis Roy, Muneendra Kumar

**Affiliations:** 1 Department of Animal Nutrition, College of Veterinary Science & Animal Husbandry, U.P. Pt. Deen Dayal Upadhyaya Pashu Chikitsa Vigyan Vishwavidyalaya Evam Go Anusandhan Sansthan, Mathura - 281 001, Uttar Pradesh, India; 2 Division of Nutrition Feed Resources and Product Technology, Central Institute for Research on Goats, Makhdoom, Farah - 281 122, Uttar Pradesh, India

**Keywords:** digestibility and quality protein maize, *in vitro*, maize varieties, methane

## Abstract

**Aim:**

To evaluate the methane generation potential and digestibility of different (normal and three high-quality protein maize [HQPM]) varieties of maize fodder with goat rumen liquor *in vitro*.

**Materials and Methods:**

Methane production potential and digestibility of different varieties of maize fodder were tested in *in vitro* gas production test. Seven varieties of maize, four normal (HTHM 5101, DHM 117, HM 5, and Shaktiman/900 M Gold), and three high-quality protein (HQPM 5, HQPM 7, and HQPM 9/Vivek) were grown in different plots under the same environmental and agro-climatic conditions. Fodders were harvested at 45-50 days of sowing, and the representative samples of fodder from different varieties of maize were collected for analysis. Dried and grinded form of these maize fodder varieties was tested for gas, methane, and digestibility using goat rumen microflora in *in vitro* gas syringes.

**Results:**

Gas production (ml/g dry matter [DM]) was highest for HM5 variety (97.66, whereas lowest for HQPM 9 variety (64.22). Gas production (ml/g degraded DM [DDM]) and methane (%) were statistically similar in different varieties of maize fodder. The methane production expressed as ml/g DM and ml/g DDM was significantly (p<0.05) highest for HM 5 (14.22 and 26.62) and lowest for DHM 117 variety (7.47 and 14.13). The *in vitro* DM digestibility (%) and *in vitro* organic matter digestibility (%) varied from 47.48 (HQPM 5) to 52.05 (HQPM 9) and 50.03 (HQPM 7) to 54.22 (HM 5), respectively.

**Conclusion:**

The present study concluded that DHM 117 maize variety fodder has lowest methane generation potential and incorporating it in the dietary regime of ruminants may contribute to lower methane production.

## Introduction

Enteric methane is normally produced during the fermentation of feeds mostly in the rumen by hydrogenotrophic methanogenic archaea, which results in the inefficient conversion of potential energy of feeds into methane that is not utilized by ruminants [1]. Methane production in the rumen represents 2-12% loss of feed energy [2] decreasing the metabolizable energy content of feeds. In addition, production of greenhouse gases from animals and their impact on climate changes are a major concern worldwide [3,4]. Methane is the second highest anthropogenic greenhouse gas after carbon dioxide, which contributes to the problems of global warming and climate change [5]. In India and other developing countries, greenhouse gases from livestock increased due to growing population of livestock and expansion of agricultural outputs in the last few decades [6,7]. Reduction in enteric methane emission enhances the efficiency of nutrient utilization and augments productivity and also reduces methane impact on global warming. Researchers are now conducting studies to identify methods of mitigating methane emissions through manipulation of the ruminant diet [8].

Feeds differ in their methane production potentiality depending on chemical composition, and plant metabolites present in them [9]. Kumar *et al*. [10] evaluated various oil cakes used in livestock feeding and found that among conventional cakes, mustard cake produced least methane. Maize is well accepted as the king of feed ingredients. About 70-80% of maize production is used for livestock feed [11]. Many varieties of maize are available for consumption by ruminants. Use of genetic selection and other biotechnological interventions have evolved various varieties of maize. Besides, altering their agronomic traits and nutritional profile, these technologies might have affected the extent of methanogenesis, and therefore, it appears that identification of varieties with lower methane generation potential might be a practical feasible option to prepare low methane producing rations for ruminants.

Keeping this in view, the present experiment was conducted to study the methane production potential of different varieties of maize fodder used in feeding of ruminants which will help in formulation of ration with low methane production that will also contribute to clean and green environment.

## Materials and Methods

### Ethical approval

The present study does not involve animal experimentation and was an *in vitro* trial. However, rumen liquor was collected as per approved norms of Institutional Animal Ethics Committee.

### Collection of feed material

Seeds of 4 normal maize varieties (HTHM 5101, DHM 117, HM 5, and Shaktiman/900M Gold) and 3 QPM varieties (high-quality protein maize [HQPM] 5, HQPM 7, and HQPM 9/Vivek) were procured from the International Maize and Wheat Centre CIMMYT Centre, New Delhi, and cultivated in the different plots of Instructional Livestock Farm Complex, U.P. Pt. Deen Dayal Upadhyaya Pashu Chikitsa Vigyan Vishwavidyalaya evam Go Anusandhan Sansthan (DUVASU), Mathura. The green fodder was ready after about 45-50 days of sowing. The representative samples of fodders were used for chemical composition and *in vitro* study.

### Processing of samples and chemical analysis

The representative samples of fodder from different varieties of maize were brought to laboratory and were dried to constant weight in hot air oven at 80°C temperature. After drying, the samples were grinded in the laboratory Wiley mill-using sieve of 2 mm diameter. The grounded samples were stored in clean, well-labeled airtight containers for further analysis. The grounded samples of different varieties of maize fodder were analyzed for proximate composition using AOAC methods [12].

### Feeding of donor animal

Rumen liquor was collected from the three adult male Barbari goats (>1 years of age) by stomach tube method. These animals were fed with 400 g concentrate pellet feed and ad lib gram straw. Water was available free choice. The rumen liquor from all the animals was collected at 0 h of feeding. After collection of rumen liquor from individual animals, they were pooled and screened through 4 layers of muslin cloths and used in preparation of media.

### *In vitro* gas production test (IVGT)

The different varieties of maize fodder were evaluated using IVGT. Substrate (0.2 g of maize fodder) was incubated for 24 h with goat rumen liquor and buffer as inoculums (30 ml) in a 100 ml capacity gas syringe as per Menke and Steingass [13]. Each set was consisting of 25 syringes (3 of each treatment, 2 standards, and 2 blanks). *Ailanthus excels* (common name Ardu) leaves have been standardized by a series of experiments for gas and methane production in our lab. These leaves were used as standard and blank was containing only media with no substrate.

### Estimation of gas production and methane

Gas production was measured as the displacement of piston of syringes. The reading of blank was subtracted to calculate gas and methane production from the substrate. After incubation at 39°C in water bath, displacement of the syringe piston indicated gas production. From the head space of each syringe, 100 µl gas was collected by purging the silicon tube and injected in gas chromatograph for the estimation of methane. It was estimated in Clarus 580 gas chromatograph from Perkin Elmer equipped with stainless steel column packed with Porapak-Q and Flame ionization detector. The standard calibration gas (Sigma gas and Services, New Delhi) consisted of 30.50% carbon dioxide, 31.16% methane, and rest is hydrogen. The flow rates for nitrogen, hydrogen, and air were 30, 30 and 30 ml/min, respectively. Temperatures of injector oven, column oven, and detector were 50°C, 40°C, and 50°C, respectively.

### Estimation of digestibility

After estimation of methane, the contents of all the syringes from each treatment were transferred separately to spoutless beakers by repeated washing with 100 ml neutral detergent solution. The flask contents were refluxed for 1 h and filtered through pre-weighed Gooch crucibles (Grade G1). The dry matter (DM) content of the residue was weighed and *in vitro* true digestibility (IVTD) of feed was calculated as follows by Van Soest and Robertson [14]:


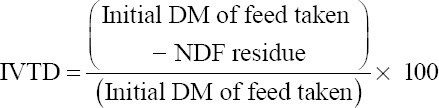


The residue was ashed at 550°C in muffle furnace and *in vitro* organic matter (OM) digestibility (IVOMD) was calculated.

### Statistical analysis

The data were analyzed by one-way ANOVA, and differences between means were compared by Duncan’s multiple range test, at a significance level of 95% as per Snedecor and Cochran methods of statistical analysis [15].

## Results

### Composition of feed samples

The proximate composition (% DM basis) of different varieties of maize fodder is presented in Table-1. The OM (%) of fodder ranged between 90.64 (HQPM 5) and 94.97 (DHM 117). Among the different maize fodder varieties, crude protein (CP) was highest for DHM 117 (10.62%) variety. The crude fiber (CF) was highest for HM 5 (23.11%) variety and was lowest for DHM 117 (16.16%). The ether extract (EE) (2.7%) was also highest for DHM 117 variety. The total ash (%) reported in different varieties varied from 5.03 (DHM 117) to 9.36 (HQPM 5). The range of nitrogen free extract (%) in different varieties of maize fodder was found between 60.03 (HQPM 5) to 65.49 (DHM 117).

**Table 1:** Proximate composition (% DM basis) of different varieties of maize fodder.

Varieties	OM	TA	CP	EE	NFE	CF
HTHM 5101	92.08	7.92	9.72	1.07	63.19	18.10
DHM117	94.97	5.03	10.62	2.70	65.49	16.16
HM5	92.16	7.84	7.29	1.68	60.08	23.11
SHAKTIMAN	91.09	8.91	9.23	1.22	62.28	18.36
HQPM 5	90.64	9.36	6.80	1.33	60.03	22.48
HQPM 7	92.12	7.88	7.21	1.46	63.05	20.40
HQPM 9	91.93	8.07	7.53	1.00	61.03	22.37

DM=Dry matter, OM=Organic matter, TA=Total ash, CP=Crude protein, EE=Ether extract, NFE=Nitrogen free extract, CF=Crude fiber, HQPM=High-quality protein maize

### In vitro fermentation of different varieties of maize green fodder

The gas production per gram DM (ml/g) was significantly (p<0.05) higher for variety HM 5 (97.66) and comparable to HQPM 5 (96.30). The gas production per gram digestible DM (ml/g DM) and methane (%) did not show significant difference among different varieties of maize (Table-2). The range of methane (%) was reported to be between 9.93 (DHM 117) and 15.90 (HQPM 9). However, a significant (p<0.05) difference was reported in net methane (ml) among different varieties of maize. The value was higher for HM 5 (2.92), and the lowest value was found in DHM 117 (1.50) variety. Similarly, significant (p<0.05) variation in methane per gram DM (ml/g DM) was also observed in different varieties of maize. It was reported to be highest for variety HM 5 (14.42) and lowest for DHM 117 (7.47) variety. However, the methane per gram digestible DM (ml/g DM) also showed significant (p<0.05) difference among the varieties being highest for variety HM 5 (26.62) and was lowest for DHM 117 (14.13) variety. The *in vitro* DM digestibility (IVDMD) of different varieties was significantly similar. The range of IVDMD (%) lies between 47.48 (HQPM 5) and 52.05 (HQPM 9). Similarly, the IVOMD of different varieties showed no significant difference among the different varieties of maize. The range of IVOMD (%) was found to be between 50.03 (HQPM 7) and 54.22 (HM 5). No significant correlation was observed between CF and digestibility of fodder of different maize varieties.

**Table 2:** *In vitro* fermentation of different varieties of maize green fodder.

Variety	Gas (ml/DM)	Gas (ml/DDM)	Methane (%)	Net methane (ml)	Methane (ml/g DM)	Methane (ml/g DDM)	DMD (%)	OMD (%)
HTHM 5101	74.40^bc^	729.17	12.63	2.06^cb^	10.26^bc^	20.03^b^	47.79	50.95
DHM117	74.30^bc^	701.40	9.93	1.50^c^	7.47^c^	14.13^c^	50.41	52.53
HM5	97.66^a^	891.73	13.55	2.92^a^	14.42^a^	26.62^a^	49.90	54.22
SHAKTIMAN	79.69^abc^	764.41	13.07	2.32^ab^	11.55^ab^	22.25^ab^	48.71	51.93
HQPM 5	96.30^ab^	888.59	12.46	2.58^ab^	12.88^ab^	23.87^ab^	47.48	54.02
HQPM 7	81.51^abc^	813.68	14.47	2.63^ab^	13.15^ab^	26.28^a^	48.31	50.03
HQPM 9	64.22^c^	615.13	15.90	2.49^ab^	12.34^ab^	23.87^ab^	52.05	51.87
Overall mean	81.15	772.01	13.15	2.36	11.73	22.44	40.24	52.22
SEM	3.30	29.99	0.44	0.11	0.57	1.07	0.56	0.49
p value	0.034	0.105	0.22	0.005	0.005	0.005	0.296	0.220

Mean bearing different superscripts differs significantly at p<0.05. DM=Dry matter, DDM=Degraded dry matter, HQPM=High-quality protein maize

## Discussion

### Composition of feed samples

The proximate compositions of different varieties of maize studied in the experiment were within the range of reported values in earlier study by Singh *et al*. [16]. Datt *et al*. [17] also observed variation in OM, CP, EE, CF, total ash, and NFE content of 10 different cultivars of maize including some varieties and their crosses. Similarly, significant (p<0.05) varietal differences in chemical composition of maize were also observed by Tolera *et al*. [18]. The nutritive value of 3 maize varieties (Sadaf, Sultan, and Sadaf white) harvested at different physiological stages was studied and variation in CP content in early (35 days) and late cut (60 days) maize fodders was found. The CP content for variety Sadaf white was found highest [19].

### In vitro fermentation of different varieties of maize green fodder

The rate of gas production varied significantly among different maize fodder varieties. The amount of gas produced from feeds depends largely on chemical composition and rate and extent of degradability of feeds [20]. The slowest rate of gas production could possibly be influenced by the fiber contents of feeds, which might be attributed to high concentrations of structural carbohydrates that are fermented at a slower rate by the rumen microorganisms. The negative correlation between fiber content and gas production is also reported in other studies [1,21]. High gas production is attributed for readily fermentable substrates [10]. In the present experiment, no significant correlation was observed between nitrogen free extract and gas production. However, Kumar *et al*. [10] observed significant (p<0.05) negative correlation (−0.747*) between gas production and neutral detergent fiber (NDF) content of the feed mixture containing different oil cakes and wheat straw. This may be because of difference in substrate (fodder) used for fermentation.

The significant difference in the net methane and methane per gram DM (ml/g DM) was observed among different maize fodder varieties. Variations in methane production among the different maize varieties may be due to variations in their chemical composition. Chemical composition of diet has also been earlier shown to have association with *in vitro* methane output [22]. However, the values for net methane (ml) produced are relatively lower, which may be due to differences in feed type, chemical composition, incubation time, and source of rumen liquor used in *in vitro* conditions.

The lowest methane production by the DHM 117 variety of maize fodder might be attributed to its high CP (10.62%) content as a significant (p<0.01) negative correlation was observed with CP content of feed with methane per gram DM (−0.912) and methane per gram digestible DM (−0.898). Lee *et al*. [23] also reported a negative correlation in methane production and CP. Likewise, Pal *et al*. [1] also reported that methane production was negatively correlated with CP, EE, and nonfibrous carbohydrate contents, and positively with NDF and acid-detergent fiber (ADF) contents. Getachew *et al*. [24] also found that ammonia, which was released by protein degradation, combines with CO_2_ the substrate for methane, and results in less methane production. It was also reported that CP produces relatively little methane [25].

The IVDMD and IVOMD (%) did not differ significantly among different varieties of maize fodder. However, a variation in the IVTD% of feeds containing different oil cakes that attributed difference in IVTD of different feed mixtures to variation in ADF content of the feeds showed a significant negative correlation (−0.721*) of ADF and IVTD (Kumar *et al.*, 2007).

## Conclusion

From the present study, it can be concluded that among the tested normal and QPM varieties of maize fodder, DHM 117 maize variety have lowest methane generation potential with no adverse effect on digestibility. So, maize fodder of DHM 117 variety can be used in the preparation of least methane producing ration.

## Authors’ Contributions

RK and VK: Designed the research work and provided the technical guidance. SV: Conducted the research work and drafted the manuscript. DR and MK: Helped in laboratory analysis. RK revised the manuscript. All authors read and approved the final manuscript.
